# Exploring the Potential of Bovine Whey Fermentation by Non-Selenized and Selenized *Enterococcus faecium* ABMC-05 for Future Functional Beverage Formulations

**DOI:** 10.3390/foods15122198

**Published:** 2026-06-18

**Authors:** Meyli Claudia Escobar-Ramírez, Emmanuel Pérez-Escalante, Luis J. Montiel-Olguín, Elizabeth Contreras-López, Luis Humberto López-Hernández, Luis Guillermo González-Olivares

**Affiliations:** 1 Centro Nacional de Investigación Disciplinaria en Fisiología y Mejoramiento Animal, Instituto Nacional de Investigaciones Forestales, Agrícolas y Pecuarias (INIFAP), Ajuchitlán, Colón 76280, Querétaro, Mexicolopez.lhumberto@inifap.gob.mx (L.H.L.-H.); 2 Área Académica de Química, Universidad Autónoma del Estado de Hidalgo, Carretera Pachuca-Tulancingo km. 4.5, Mineral de la Reforma 42184, Hidalgo, Mexico; emmanuel_perez@uaeh.edu.mx (E.P.-E.);

**Keywords:** probiotic, selenium, whey protein hydrolysates

## Abstract

This study aimed to evaluate whey fermentation with *Enterococcus faecium* ABMC-05 to obtain a product with three functional ingredients: probiotic strains, selenium enrichment, and hydrolysates with antioxidant and antihypertensive properties. Whey, with and without sodium selenite (184 mg/mL), was fermented by *Enterococcus faecium* ABMC-05 and analyzed for 120 h. Free amino groups (TNBS), protein hydrolysis (Tris-Glycine-SDS-PAGE), lower molecular protein fractions (Tris-Tricine-SDS-PAGE and SEC-HPLC), antioxidant activity (DPPH and FRAP), and antihypertensive activity (ACE inhibition) were determined. The results showed that selenium accumulation in *Enterococcus faecium* ABMC-05 gradually increased during fermentation, reaching 2.21 µg Se/Log CFU. This was associated with a delay in the initial stage of bacterial growth and a greater release of free amino groups. Partial hydrolysis of serum β-lactoglobulin was observed by SDS-PAGE and confirmed by HPLC only in the fermentation without selenium. The levels of inhibition of the DPPH radical decreased during fermentation in both systems, while FRAP remained unchanged during the fermentation time in the selenium system. In contrast, ACE inhibitory activity increased to 53% at 120 h of fermentation in the selenium system. Therefore, the combination of the three functional ingredients may enhance bioactivity and serve as an alternative in functional dairy foods.

## 1. Introduction

The functional foods market, a global phenomenon, has grown rapidly in importance, reaching an estimated USD 398.81 billion in 2025 and is projected to reach USD 983.17 billion by 2034 [1]. Functional foods are a broad category encompassing both natural and processed foods that, when consumed at efficacious levels, can contribute to health beyond essential nutrition. They are a vital part of this market and must also be safe and accessible [2].

Dairy products are at the forefront of matrices used in functional food development, followed by bakery products and cereals [2,3]. This trend is driven by consumer attitudes toward price, with higher prices often associated with higher-quality products or viewed as a purchase sacrifice [3,4]. However, novel trends are emerging to cater to all types of economies, with a diverse range of products such as beverages, shakes, protein bars, cookies, gummies, and toffees addressing health claims related to obesity, weight management, diabetes, and cardiovascular diseases [5,6]. Among the most popular functional ingredients added to food to reduce the risk of these pathologies are vitamins, dietary fiber, prebiotics, and probiotics. These microorganisms, recognized as functional a few years ago, can positively influence human health and well-being, offering a promising outlook [7,8,9]. Lactic acid bacteria (LAB), commonly found in these products and represented by the genera *Lactobacillus* and *Bifidobacterium*, have been shown to be effective in treating a range of health conditions [10,11,12]. In addition, they also exhibit hypocholesterolemic, antimutagenic, antiosteoporotic, antihypertensive, and immunomodulatory effects [13,14].

Despite *Lactobacillus* and *Bifidobacterium* species being the most widely tested probiotic genera, other lactic acid bacteria have been considered potential probiotics in recent years. In this context, *Enterococcus* spp. have been established as excellent probiotic sources, as these species have demonstrated safety in virulence and antibiotic-resistance assays [14,15,16,17]. Specifically, some *Enterococcus faecium* strains have achieved this recognition due to the promising results obtained in recent years. For example, the *Enterococcus faecium* OV3-6 strain maintained a survival rate of 88.16% and 94.33% under simulated gastrointestinal conditions [18]. Also, the strain could regulate the secretion of pro- and anti-inflammatory cytokines in cell lines, inhibit the adhesion of pathogenic bacteria, and produce an antimicrobial peptide. Similarly, *Enterococcus faecium* MG5232 exhibited approximately 66% autoaggregation, a parameter associated with in vitro intestinal adhesion that contributes to the dissemination of harmful bacteria into the intestine [19]. In addition, *Enterococcus faecium* SF68 can reduce intestinal inflammatory symptoms through arginine deiminase, which exerts anti-inflammatory and immunomodulatory effects by inhibiting the NF-κB and JNK (AP-1) pathways [20].

Other functional food ingredients recognized a few years ago include bioactive peptides, sequences of 2 to 20 amino acids derived from the hydrolysis of animal or plant proteins. These could confer health benefits, especially in preventing non-communicable diseases. The bioactivities of these short amino acid sequences include antioxidant, antihypertensive, hypocholesterolemic, immunomodulatory, antimicrobial, antithrombotic, and anticancer activities [21,22,23].

As these functional protein fractions are inactive in their native source, they have been obtained using two main procedures. The first uses isolated enzymes such as alcalase, flavourzyme, pepsin, or pancreatin, and the second uses some lactic acid bacteria with probiotic or non-probiotic properties, exploiting their proteolytic systems for nitrogen metabolism [24,25,26]. In this sense, milk fermentation is an excellent alternative for bioactive peptide production because caseins, α-lactalbumin, and β-lactoglobulin consumed by lactic acid bacteria during their growth can be converted into bioactive peptides. For the specific case of whey, research has shown that its hydrolysis produces peptides that inhibit ACE, DPP-IV, and thrombin, exhibit radical-scavenging properties, or suppress viral gene expression [27,28].

On the other hand, selenium has also been considered a potential functional food ingredient due to its roles in immune function, antioxidant defense, and cancer chemoprevention [29,30]. Thus, novel trends in food and beverage formulations that incorporate selenium have emerged over the past few years, offering an alternative for populations with limited access to selenium-enriched foods due to geographic conditions [29,31]. In this sense, the primary approach to food selenium fortification has been the incorporation of selenized microorganisms, which can convert inorganic selenium species into selenonanoparticles with antimicrobial activity or into selenoamino acids with better intestinal bioavailability [32,33,34]. Thus, this work aimed to assess the potential of whey fermentation with *Enterococcus faecium* ABMC-05 to produce a product containing three functional ingredients: a probiotic strain, selenium enrichment, and hydrolysates with antioxidant and antihypertensive properties, which may be useful in future beverage formulations.

## 2. Materials and Methods

### 2.1. Strain Acquisition and Propagation Conditions

The strain *Enterococcus faecium* ABMC-05 was previously isolated from a traditional Mexican beverage known as “tepache” [35,36]. It was registered in the NCBI database with accession number NCBI-OL413240.

Before whey fermentation, the strain was propagated as follows: from a bacterial stock stored in glycerol at −20 °C, three subcultures were performed in MRS broth at 37 °C for 12 h. Subsequently, 0.10 mL of the fresh MRS subculture was inoculated into 10 mL of fermentation culture medium and incubated at 37 °C for 24 h. The fermentation culture medium consisted of 10% (*w*/*v*) WPC-80 and 1% (*w*/*v*) glucose and was thermally treated at 75 °C for 15 min. After the bacteria were subcultured three times, the culture was stored at 4 °C and used as a starter culture.

### 2.2. Fermentation Process

Two fermentation systems were proposed. The first was the control medium, WPC-80 with glucose (as previously described), and the second was the same medium supplemented with 184 mg/L Na_2_SeO_3_, corresponding to the minimum inhibitory concentration for the microorganism used [35]. Fermentation was carried out at 37 °C for 120 h, with samples collected every 12 h. Samples were analyzed for pH changes and viable counts by plate counting on MRS agar using the microdrop technique. Finally, after analysis, the supernatants from each sample were collected by centrifugation at 10,000 rpm for 15 min at 4 °C and stored at −18 °C.

### 2.3. Quantification of Total Selenium by Inductively Coupled Plasma Atomic Emission Spectroscopy (ICP-OES)

Biomass produced by *Enterococcus faecium* ABMC-05 during each sampling time was obtained from 1 mL of fermented whey, which was centrifuged under the conditions described before. In addition, cells were washed twice with 0.100 mL of dithiothreitol (0.3%) and then centrifuged under the same conditions. Supernatants from both washes were combined, and 1 mL was digested in 10 mL of concentrated nitric acid using microwave heating (175 °C/5.5 min, 175 → 180 °C/4.5 min; 110 psi). Finally, after digestion, the resulting solution was brought to a final volume of 25 mL, and selenium was quantified at 166 nm using an external standard calibration curve spanning 0–5 mg/L (Na_2_SeO_3_) [37]. The difference between each sampling time and the selenium concentration at the start of fermentation was calculated to determine the selenium absorbed.

### 2.4. Free Amino Groups Quantification

To determine the hydrolysis grade, supernatants from the fermentation process were subjected to the 2,4,6-trinitrobenzene sulfonic acid (TNBS) method proposed by Adler-Nissen [38]. In brief, 0.250 mL was mixed with 2 mL of phosphate buffer (0.21 M, pH = 8.2) and 2 mL of TNBS solution at 0.1% (*v*/*v*) dissolved in phosphate buffer. The reaction was carried out for 1 h at 50 °C and stopped by the addition of 4 mL of HCl (0.1 N). Finally, absorbance was recorded at 340 nm, and the concentration of free amino groups was determined from a glycine calibration curve (0–200 mg/L).

### 2.5. Electrophoretic Analysis

To visualize changes in protein hydrolysis, Tris-Glycine-SDS-PAGE and Tris-Tricine-SDS-PAGE were performed [39,40]. For glycine electrophoresis, a separation gel at 15%T and a concentration gel at 4%T were prepared from an acrylamide/bis-acrylamide solution (40%, 37.5:1, 2.7% crosslinker, Bio-Rad, Hercules, CA, USA). Sample running was conducted at 200 V until the samples reached the front of the gel. For tricine electrophoresis, the separation gel was 16.5%T, while the concentration gel was 4%T, both prepared from a 40% acrylamide/bis-acrylamide solution (19:1, 5% crosslinker, Bio-Rad). The running operation was conducted at 30 V for 2 h, then at 95 V until the samples migrated across the entire concentration gel. Before electrophoretic separation, samples were standardized to a soluble protein content of 150 mg/L for glycine and 250 mg/L for tricine-SDS-PAGE. Finally, a gel image was obtained through the Gel Doc EZ system (Bio-Rad).

### 2.6. Size Exclusion Chromatography (SEC-HPLC)

Size-exclusion chromatography was performed to identify changes in whey proteins and in lower-molecular-weight protein fractions. The methodology proposed by Pérez-Escalante et al. [41] was followed. Samples were standardized to a soluble protein content of 250 mg/L, and 20 µL were injected into an SRT-SEC-150 column (SEPAX Technologies, Inc., Newark, DE, USA) (300 mm × 7.6 mm × 5 µm). Separation was performed under isocratic flux (0.5 mL/min for 60 min) using 0.1 M phosphate buffer (pH 6.8) as the mobile phase. Detection was performed at 220 nm, and molecular weights were estimated from an external standard calibration curve where solutions at 1 mg/mL of urease from Canavalia ensiformis (544.62 kDa), bovine serum albumin (66.34 kDa), ovalbumin (45 kDa), β-lactoglobulin (18.40 kDa), aprotinin (6.5 kDa), and (Glu1)-Fibrinopeptide B (1.57 kDa) were used as molecular weight standards.

### 2.7. Antioxidant Activity Determination

Two antioxidant activity assays were performed, as described by Ramírez-Godínez et al. [42]. The first was the DPPH assay, in which 2.9 mL of 2,2-diphenyl-1-picrylhydrazyl (DPPH) at 0.1 mM dissolved in methanol was mixed with 0.1 mL of fermentation supernatant. The reaction was carried out in the dark and incubated at room temperature for 50 min. The absorbance was measured at 515 nm, with methanol as the blank. At the same time, the control was measured by substituting 0.1 mL of the sample with methanol. The remaining DPPH percentage was calculated using the following equation: Remaining DPPH (%) = ((sample absorbance − blank absorbance)/control absorbance) × 100. Finally, results were reported as Trolox mg eq./100 mL of sample, based on an external standard curve at 0, 8.3, 16.6, 25, and 33.3 µM.

The second antioxidant test was performed through the FRAP method. Thus, 1 mL of the FRAP reagent, composed of acetate buffer 0.3 M (pH 3.6), 2,4,6-Tri(2-pyridyl)-s-triazine (TPTZ) at 10 mM dissolved in HCl (40 mM), and FeCl_3_·6H_2_O at 20 mM in a volume ratio 10:1:1, was mixed with 0.250 mL of the fermentation samples and brought to a final volume of 10 mL with deionized water. The reaction was performed in darkness at 37 °C for 15 min, and absorbance was measured at 593 nm, with a blank prepared by mixing 9 mL of deionized water with 1 mL of the FRAP reagent. Results were expressed as Fe^2+^ mg eq./100 mL of sample, based on an external standard curve performed at 0–0.03 mM Fe^2+^ from FeSO_4_·7H_2_O.

### 2.8. Antihypertensive Capacity Determination

The antihypertensive capacity of fermentation supernatant was evaluated through Angiotensin-Converting Enzyme (ACE) inhibition. The analysis was based on the proposal by Hussein et al. [43] with some modifications. Two reaction systems were assessed: the first, denominated the positive control (A100), was composed of 80 µL of saline borate buffer (0.05 M, pH 8.2), which contained NaCl at 0.3 M, 200 µL of hippuryl–histidyl–leucine (HHL) as a substrate at 5 mM dissolved in saline borate buffer, and 20 µL of ACE (SIGMA, A6778-.1UN) from rabbit lung at 0.1 U/mL dissolved in saline borate buffer. The second system, the sampling system (Am), contained the same volumes of substrate and enzyme, with the hydrolysates substituting for 80 µL of saline borate buffer. Both systems were incubated for 80 min at 37 °C, and the reaction was stopped by adding 250 µL of 0.1 M HCl. Subsequently, the reaction mixture was combined with 1.7 mL of ethyl acetate and mixed three times to extract hippuric acid. From the last mixture, 800 µL of the organic layer was separated, and the solvent was removed by heating at 80 °C for 1 h. Afterward, the remaining residue was dissolved in 500 µL of deionized water, then mixed with pyridine (300 µL) and benzene sulfonyl chloride (150 µL). Finally, absorbance was recorded at 410 nm using water as a blank, and the ACE inhibition was calculated from the following equation: ACE inhibition (%) = ((A100 − Am)/A100) × 100.

### 2.9. Statistical Analysis

Data were analyzed using a linear mixed model, with fermentation system, time, and their interaction as fixed effects and id as a random effect. A repeated-measures design with an autoregressive covariance structure AR(1) was used to account for correlation among observations over time. Least-squares means were compared using Tukey’s adjustment, and statistical significance was set at *p* < 0.05. All analyses were performed in triplicate except for ACE inhibition, which was performed in duplicate. Model diagnostics were conducted using PROC UNIVARIATE to confirm the normality and homoscedasticity of residuals. All statistical analyses were performed using SAS software version 9.4 (SAS Institute Inc., Cary, NC, USA).

## 3. Results and Discussion

### 3.1. Enterococcus faecium ABMC-05 Growth in Whey With and Without Selenium

**Figure 1:**
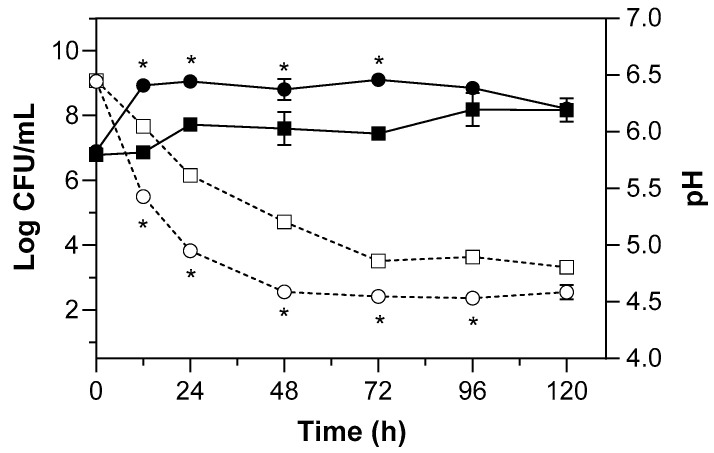
Changes in viability and pH during the whey fermentation with *E. faecium* ABMC-05. ● viability WPC-80, ■ viability WPC-80 + Na_2_SeO_3_, ○ pH WPC-80, □ pH WPC-80 + Na_2_SeO_3_. * Denotes significant differences between WPC-80 and WPC-80 + Na_2_SeO_3_ at the same fermentation times (*p* < 0.05).

*E. faecium* ABMC-05 could grow in both selenium-enriched and non-enriched media. Changes in its growth and pH are shown in Figure 1.

The growth of *E. faecium* ABMC-05 was significantly affected (*p* < 0.05) by interactions between the culture medium (WPC and WPC-Se) and fermentation time. *E. faecium* ABMC-05 grew faster in a culture medium without selenium, as the adaptation phase was not observed, unlike the medium with selenium, in which the adaptation phase was observed in the first 12 h. However, in both fermentation systems, the strain reached its maximum growth at 24 h, with a prolonged stationary phase lasting until 96 h, and reached a cell concentration of 8 log CFU/mL at the end of fermentation (120 h), without reaching the death phase.

Moreover, as observed in other studies, selenium decreased microbial growth, displacing the growth curve by 2 log cycles during the first 12 h and by 1 log cycle thereafter compared with the control medium. In the presence of selenium, lactic acid bacteria exhibit decreased growth because they must obtain their nutritional requirements from the culture medium while simultaneously investing energy in selenium biotransformation as a detoxification mechanism [37,44,45,46]. In this context, when bacteria are exposed to elevated selenite concentrations, detoxification pathways may be activated to limit the intracellular accumulation of toxic selenium species. Consequently, selenite can be reduced to form organic selenium compounds.

Nonetheless, this study demonstrated that whey is an excellent growth medium for *E. faecium* ABMC-05, even at high concentrations of sodium selenite. In contrast, the same strain showed no increase in MRS broth under the same sodium selenite concentration [36]. Additionally, other studies have demonstrated that selenium promotes the growth of lactic acid bacteria only at low doses [45,47,48,49,50,51,52,53], reflecting whey’s feasibility for selenized LAB growth.

The observed growth could be associated with amino acid availability in the culture medium and the strain’s nitrogen requirements. Regarding the latter, *Enterococcus* spp. show auxotrophies in dairy matrices for nine primary amino acids (Arg, Ile, Phe, Leu, Thr, Trp, His, Met, Val), which can be acquired from whey, either as free amino acids availability or through α-lactalbumin and β-lactoglobulin hydrolysis when the enterococcal proteolytic system is activated [54,55,56].

pH changes are closely related to bacterial metabolism. Thus, the acidification of *E. faecium* ABMC-05 was also significantly modified (*p* < 0.05) by the presence of selenium during fermentation. The strain in whey medium lowered the pH from 6.5 to 4.5 in 48 h, while in the medium with selenium, a pH of 4.8 was reached after 72 h; in both cases, once the lower pH was achieved, it remained constant during the rest of fermentation, as has been reported previously for the strain *E. faecium* CCDM 922A [52]. In addition, some reports have found that *E. faecium* exhibits weak acidification when growing in milk or whey, reaching pH values near or slightly below 5 [57,58], consistent with this observation in this study.

### 3.2. Selenium Accumulation

The selenium content in the biomass and the fermentation supernatant was evaluated to determine its uptake during growth in bovine whey enriched with sodium selenite at a minimum inhibitory concentration. The results are shown in Table 1.

As expected, the selenium content decreased in the supernatants. At the same time, it was increased in the cells, especially during the first 72 h, because after that time, selenium uptake remained constant, as its accumulation showed no significant differences (Table 1), achieving selenium uptakes of 52–59% and selenium accumulations around 2 µg Se/log CFU. This behavior suggests that the strain’s selenium accumulation in whey depends on the fermentation time. However, it is also consistent with the stationary phase and medium acidification discussed above.

Results also indicate that selenium accumulation can vary among strains, as other LAB grown in milk enriched with sodium selenite showed that fermentation time did not influence bioaccumulation [48,59]. Moreover, the present study demonstrates that the selected culture medium could affect selenium accumulation because the same strain could accumulate between 3.35 and 4.82 µg Se/log CFU in MRS broth from 72 to 120 h [36], revealing that higher selenium bioaccumulation is carried out during the prolonged stationary phase, as in whey as MRS medium.

Although *E. faecium* ABMC-05 showed lower selenium bioaccumulation in whey, this fermentation process generates a product that could be consumed directly. Indeed, although an animal model must be used to assess food safety in future studies, the available toxicity data suggest that this fermented product could be incorporated into dairy functional products. A recommended daily intake of selenium has been established at 50–70 µg/day for adults, with a maximum tolerable intake of 255 µg/day [60,61]. Therefore, based on the in vitro accumulation levels observed, an aliquot of 1.6–2.2 mL of the fermented product would theoretically contain the recommended daily intake of selenium. However, in vivo bioavailability studies would be essential to confirm its safety and efficacy before human consumption.

On the other hand, the strain has demonstrated the ability to biotransform inorganic selenium into selenocysteine in MRS medium [35,48,49], suggesting that although absorption was lower in bovine whey, biosynthesis of this selenoamino acid may still be possible. However, a more detailed study is needed to determine the specific selenium species produced and their concentrations.

### 3.3. Proteolytic Profile

#### 3.3.1. Hydrolysis Degree by Following the Free Amino Groups

**Figure 2:**
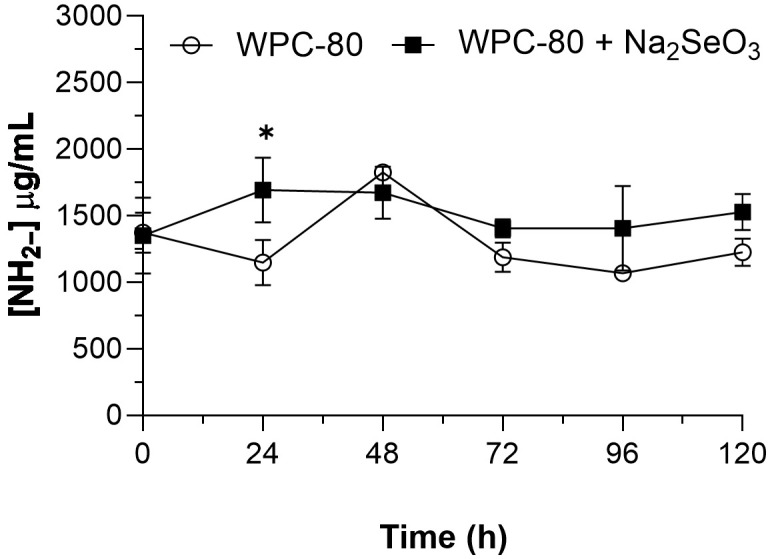
Free amino group production by *E. faecium* ABMC-05 during its growth in whey with and without selenium. * Denotes fermentation times with significant differences (*p* < 0.05).

The degree of hydrolysis is a parameter used to quantify protein fractionation. It also represents the cleavage ratio of the peptide bond in the native protein. Therefore, this analysis was conducted to assess the impact of selenium on hydrolysis. The results are shown in Figure 2.

As previously observed for viable count and pH changes, the enriched medium containing sodium selenite also altered the degree of hydrolysis relative to the control. A significant interaction between culture medium and fermentation time (*p* < 0.05) was also observed. The concentration of free amino groups at 24 h differed significantly (*p* < 0.05) from that at other fermentation times across both systems.

At 24 h, the control medium with whey showed a significant decrease (*p* < 0.05), suggesting that the enterococci had low nitrogen requirements and used only a portion of the nitrogen available in the whey to achieve faster, exponential growth. At 48 h, the highest hydrolysis was observed, indicating that the proteolytic system was most active between 24 and 48 h. This latter behavior may correlate with changes in the viable count, in which the microorganism activates its proteolytic system to meet nitrogen requirements from whey proteins and prolongs its stationary phase, thus avoiding cell death.

Subsequently, consumption of the released whey fractions became evident from 72 h onward, accompanied by a decrease in free amino group concentration, which remained unchanged until the end of fermentation, coinciding with the stationary phase of the microorganism.

In the medium containing sodium selenite, faster hydrolysis was observed at 24 and 48 h, corresponding to the exponential phase and the first extension of the stationary phase. The results showed that an additional nitrogen source from whey protein hydrolysis was required when the initial available concentration was insufficient to support the growth of *E. faecium* ABMC-05 in the presence of selenium, possibly due to selenium-induced stress. Then, at 72 h, the first decrease was observed, indicating consumption of peptides generated earlier, thereby extending the stationary phase and preventing cell death. At subsequent time points, peptide uptake was observed and was associated with a novel form of stationary-phase extension.

Few studies have examined the enterococcal proteolytic system to date. Nonetheless, this genus harbors a Clp complex capable of hydrolyzing α-lactalbumin and β-lactoglobulin, thereby generating oligopeptides that are subsequently taken up by the cell via the Opp, Dpp, and DptT transport systems. Afterward, these peptides are hydrolyzed again by proteolytic enzymes (Pep O, Pep F, Pep V, Pep C, Pep A, Pep Q) into smaller peptides and essential amino acids to meet nitrogen requirements [62,63]. In addition, it has been observed that peptidic fractions not useful for bacterial metabolism are excreted into the medium, particularly during the final steps of proteolytic systems, coinciding with the end of the exponential growth phase [64,65,66]. Therefore, the increase in free amino groups at 24–48 h in the selenium-enriched system is primarily due to the activation of the Clp complex in response to the increased nitrogen demand resulting from the presence of selenium. Conversely, the decrease after 72 h may be due to the peptide transport system.

#### 3.3.2. Monitoring Whey Protein Proteolysis Using SDS-PAGE and Size-Exclusion Chromatography

**Figure 3:**
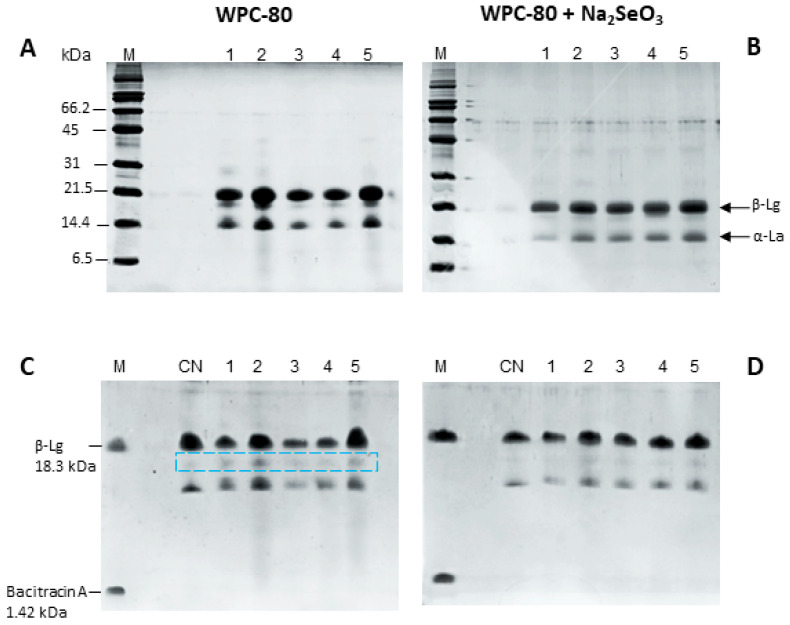
Protein and lower protein fractions separation by Tris-Glycine-SDS-PAGE (**A**,**B**) and Tris-Tricine-SDS-PAGE (**C**,**D**) from whey fermentation by *E. faecium* ABMC-05. CN: Negative control; samples 1–5 correspond to 24, 48, 72, 96, and 120 h of fermentation, respectively. M: Broad range protein marker. β-Lg: β-lactoglobulin. α-La: α-lactalbumin. The blue dashed lines indicate the hydrolysis of whey proteins.

Tris-Glycine-SDS-PAGE, as well as Tris-Tricine-SDS-PAGE, for the control and selenium-enriched whey systems are shown in Figure 3. Both electrophoresis experiments showed limited proteolysis for both proposed systems.

As discussed, few studies on the enterococcal proteolytic system have been reported; however, some studies suggest that the proteolytic activity of *E. faecium* is generally lower than that of other lactic acid bacteria [57,67], reaching concentrations of 230–970 mg/L of tyrosine released at 72 h of fermentation in milk [58]. In addition, *E. faecalis* exhibited higher proteolysis (2000–2500 mg/L) during milk fermentation [68], but this effect was significant only up to the stationary phase (24 h).

Despite the determination of free amino groups, which indicated protein hydrolysis, electrophoresis analysis generally showed limited degradation of whey proteins in the control system. The Tris-Glycine-SDS-PAGE showed increased band intensity in the region corresponding to molecular weights below 14 kDa at 48 and 120 h of fermentation, suggesting the formation of low-molecular-weight hydrolysates. In contrast, this behavior was not observed in the selenium-enriched system, where minimal hydrolysis of β-lactoglobulin and α-lactalbumin was detected. Furthermore, Tris-Tricine-SDS-PAGE showed that *E. faecium* ABMC-05 preferentially hydrolyzed β-lactoglobulin over α-lactalbumin in both systems, generating protein fractions with molecular weights between 18.3 and 14 kDa. These fractions appeared less abundant in the selenium-enriched system, suggesting that they were further metabolized by the strain, possibly as part of the adaptive response to selenium-induced stress discussed previously.

Studies on *Enterococcus* spp. suggest the presence of proteinases for the partial degradation of β-lactoglobulin and α-lactalbumin [69]. Only metalloproteases have been identified in *E. faecium* strains, whereas in *E. faecalis*, both metalloproteases and serine proteases have been identified [70,71,72,73]. This could explain the differences observed in this study, in which β-lactoglobulin was preferentially proteolyzed compared with the α-lactalbumin preference of *E. faecalis* strains.

**Figure 4:**
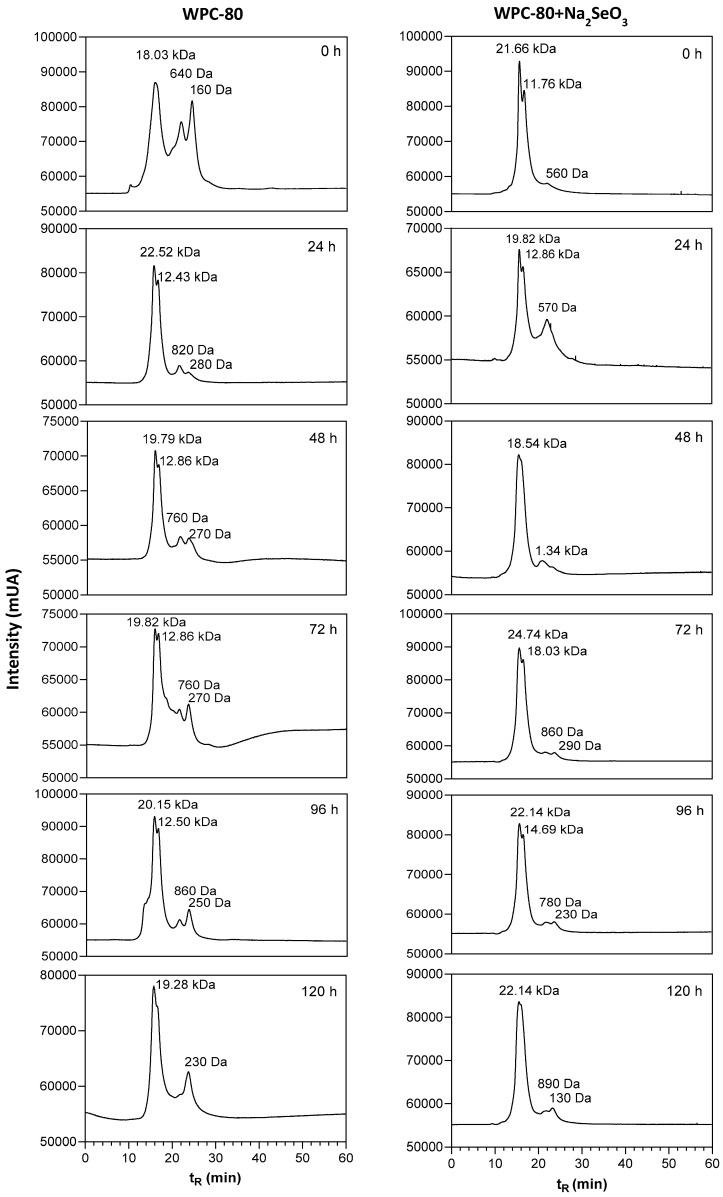
Peptides profile by size exclusion chromatography for both fermentation systems, every 24 h during 120 h. t_R_ = retention time.

To complement the SDS-PAGE analysis, samples were separated by size-exclusion chromatography. The results confirm the previously observed electrophoretic profile, in which the signal for β-lactoglobulin (approximately 18 kDa) diminished in all fermentations in both systems, with the control showing the highest signal (Figure 4). Additionally, chromatographic analysis revealed protein fractions with molecular weights between 160 and 640 Da in the control systems, confirming the growth behavior of *E. faecium* ABMC-05 and the previously discussed activation of its proteolytic system. Moreover, the chromatograms showed partial β-lactoglobulin degradation, as evidenced by an unfolding signal corresponding to a protein fraction of approximately 12 kDa. In addition, separation enabled observation of consumption of the 160–640 Da fractions in the first 24 h and of the release, consumption, and accumulation of fractions between 200 and 800 Da, with higher concentrations at the end of fermentation, especially for the 200 Da fraction.

In the case of the enriched selenium system, the β-lactoglobulin unfolding signal was also exhibited. The main differences relative to the control were an increase in the fraction with a molecular weight of 570 Da at 24 h, which coincided with an increase in free amino groups, and a lower accumulation of protein fractions between 200 and 800 Da. As previously discussed, *Enterococcus* spp. exhibits a slow whey protein hydrolysis [71]. However, it is evident that the presence of selenium further reduced this activity, as factors such as metabolic stress [73,74] and possible interactions of this trace element with metalloproteases [75] reduced their activity and, consequently, peptide accumulation.

### 3.4. Antioxidant Evaluation for Whey Fermentation Systems

**Figure 5:**
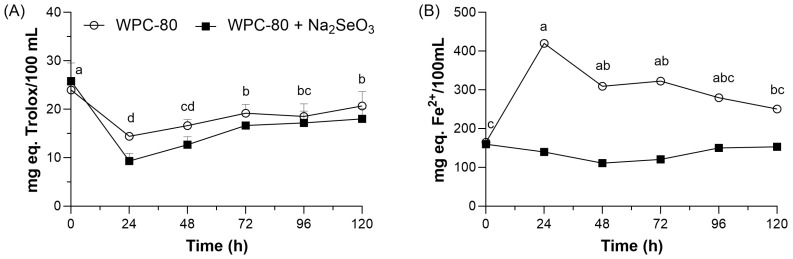
Antioxidant properties from whey fermentation by *E. faecium* ABMC-05 determined by DPPH (**A**) and FRAP (**B**). (**A**) Different letters indicate significant differences in fermentation times (*p* < 0.05). (**B**) Different letters indicate a significant interaction between culture medium and fermentation time (*p* < 0.05); only WPC-80 showed significant changes over time.

The antioxidant and antihypertensive properties were assessed throughout the fermentation process. Figure 5 shows the results for antioxidant capacity, based on radical-scavenging ability and ferric-reducing power, measured using the DPPH and FRAP methods.

The radical scavenging activity showed the same pattern in both fermentation systems. A significant decrease (*p* < 0.05) was observed after 24 h of fermentation, followed by a gradual and consistent recovery from 72 h through the end of the process. Although the selenium-enriched system exhibited lower DPPH scavenging activity throughout most of the fermentation period, both systems reached comparable antioxidant activity levels at the end of fermentation.

The last behavior is associated with the previously discussed proteolytic profile and with the commonly reported molecular weights of antioxidant peptides, which range from 500 to 1500 Da [76,77]. Consistent with these reports, size-exclusion chromatography revealed the presence of whey protein hydrolysate fractions within the 500–600 Da range in both fermentation systems. Although the peptide sequences were not identified in the present study, these low-molecular-weight fractions may have contributed to the radical-scavenging activity observed during fermentation.

In addition, the initial size-exclusion chromatography profile suggests that whey protein hydrolysate concentration was not the sole factor affecting antioxidant activity, as both fermentation systems exhibited similar DPPH scavenging capacities despite the higher signal intensity of the 500–600 Da fractions observed in the control medium. A similar pattern was observed from 72 h until the end of fermentation, indicating that, in addition to fermentation time, bioactivity is closely linked to the amino acid sequence rather than to the protein fraction concentration. In this regard, antioxidant peptides derived from whey proteins, particularly from β-lactoglobulin hydrolysis, have been reported to contain amino acids such as tryptophan, threonine, tyrosine, valine, and lysine [78]. The proteolytic profiles obtained by Tris-Tricine-SDS-PAGE and size-exclusion chromatography indicated that β-lactoglobulin was utilized during fermentation by *E. faecium* ABMC-05, suggesting the potential generation of hydrolysates with antioxidant properties. However, more specialized analyses should be carried out to identify specific peptide sequences released by *E. faecium* ABMC-05.

On the other hand, the FRAP assay showed that the iron-reducing activity of whey fermented by *E. faecium* ABMC-05 in the presence of selenium remained unchanged throughout fermentation, despite higher levels of free amino groups observed in this system compared with the control. This finding suggests that the whey protein hydrolysates generated under these conditions did not substantially enhance the iron-reducing capacity of the fermentation medium. The structural characteristics of peptides with iron-reducing potential may explain this behavior. According to Zou et al. [79] and Nwachukwu & Aluko [80], antioxidant peptides with radical scavenging and iron-reducing properties share some similitudes as they display hydrophobic amino acids in their structure like isoleucine, proline, glycine, or methionine. In addition, the presence of lysine and the tyrosine:tryptophan ratio have been reported as important structural features influencing iron-reducing capacity [78].

Another difference between radical-scavenging and iron-reducing peptides is their size: peptides that stabilize DPPH radical-scavenging activity are predominantly tetra- or pentapeptides with molecular weights below 1 kDa. In contrast, iron-reducing peptides are generally composed of 8–10 amino acids with molecular weights greater than 1000 Da [79,80]. Thus, the FRAP results obtained in this study suggest a possible relationship between fraction size and concentration of whey protein hydrolysates during fermentation. In this regard, size-exclusion chromatography analysis showed a higher signal intensity for fractions with molecular weights close to 1000 Da in the control medium than in the selenium-enriched medium. Furthermore, selenium-induced changes in the proteolytic system of *E. faecium* ABMC-05 may have influenced the generation of whey protein hydrolysates and, consequently, their iron-reducing activity, as lactic acid bacteria are known to modify their metabolism in response to selenium exposure [45].

As discussed, the use of *Enterococcus* spp. as starter cultures has been limited by food safety concerns. However, its application to fermented products has gained relevance in recent years, particularly due to the proteolytic activity of these products and their potential to generate bioactive protein hydrolysates. Bhagwat and Annapure [81] assessed the antioxidant capacity of supernatants from *Enterococcus* strains grown on MRS medium and found DPPH inhibition ratios of 30–50% and FRAP reduction; however, only two strains showed activity comparable to Trolox. Similarly, Krausova et al. [52] and Pieniz et al. [82] evaluated the antioxidant capacity of *Enterococcus* strains cultivated in M17 and BHI media, respectively, using the DPPH assay. These authors reported DPPH inhibition values ranging from 1.6% to 4% and IC_50_ values between 2.41 and 5.02 µg/mL, demonstrating strain-dependent variability of the antioxidant potential within this genus.

Other studies assessing supernatants from milk or whey fermentation by lactic acid bacteria have been reported by Graham et al. [83] and Lee et al. [84]. The first research group found antioxidant activity, stabilizing the DPPH radical between 42 and 271 µM (Trolox equivalents) and, in the FRAP test, between 71 and 273 µM in *E. faecalis* fermentation. In contrast, the second research group found antioxidant activity of approximately 20 mM ascorbic acid equivalents in the DPPH assay for *L. paracasei* DK209.

In the present study, the inhibition percentages for the DPPH radical oscillated between 26 and 37% for whey without selenium and between 16 and 32% for the selenium-enriched medium, which corresponded to a Trolox equivalent concentration of 57.6–82.6 and 37.2–72 µmol/100 mL of hydrolysate for control and enriched medium, respectively. In addition, FRAP values ranged from 150.7 to 413.6 mg Fe^2+^ equivalents/100 mL of hydrolysate. These results indicate that whey fermentation with *E. faecium* ABMC-05 generated hydrolysates with antioxidant activity comparable to or greater than that reported for some *Enterococcus* strains. Furthermore, the iron-reducing capacity observed in the fermented hydrolysates suggests the formation of bioactive compounds during fermentation. However, the specific compounds responsible for these activities were not identified and should be characterized in future studies.

When the antioxidant activity of whey fermented by *L. paracasei* DK209 is compared with that reported in the present study, the lactobacilli fermentation shows higher bioactivity; however, the antioxidant activity measured in the present study was derived from whey protein hydrolysis by *E. faecium* ABMC-05, as the analyzed supernatants were cell-free. Nevertheless, the overall antioxidant potential of the fermented product may not be fully reflected by these in vitro assays alone. Previous studies have shown that *E. faecium* ABMC-05 is capable of transforming inorganic selenium into organic selenium compounds, including selenocysteine, a selenium-containing amino acid with well-documented antioxidant properties [35,36,85,86]. Indeed, a similar study by Krausova et al. [52] reported lower antioxidant potential when *Enterococcus faecium* CCDM 922A fermentation supernatants were analyzed using an in vitro assay. However, when selenized bacteria were administered to rats, the animals’ antioxidant status improved [73], with selenocysteine as the predominant selenium species. Therefore, although the selenium species produced during fermentation were not characterized in the present study, these findings suggest that *E. faecium* ABMC-05 could be a promising microorganism for the development of functional fermented dairy products. However, further studies are required to characterize its selenium species and evaluate its biological effects in vivo.

### 3.5. ACE Inhibition Evaluation for Whey Fermentation Systems

**Figure 6:**
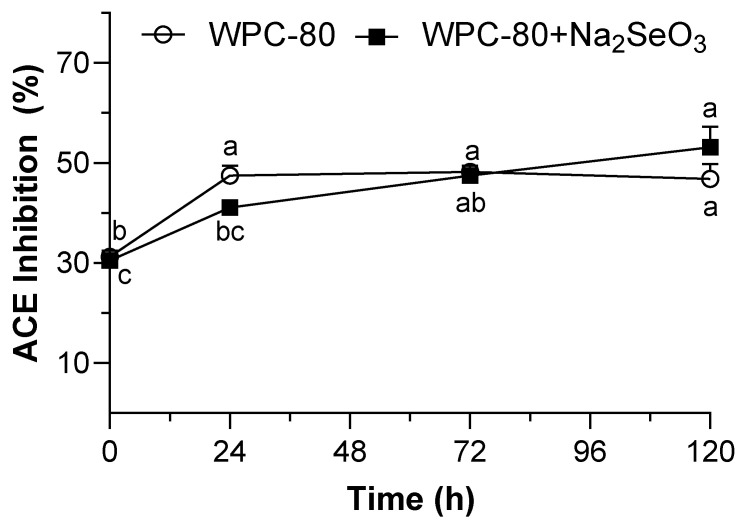
Evaluation of ACE inhibition from whey fermentation by *E. faecium* ABMC-05. ^a,b,c^ Different letters indicate significant differences (*p* < 0.05) among fermentation times within the same system.

Angiotensin-converting enzyme inhibition is widely recognized as an effective therapeutic strategy for the management of hypertension. The results of the present study showed that ACE inhibitory activity increased significantly (*p* < 0.05) during fermentation by *E. faecium* ABMC-05 in both systems (Figure 6). However, distinct inhibition patterns were observed. In the selenium-enriched system, ACE inhibition increased progressively throughout fermentation, reaching 53% at 120 h. In contrast, the control system showed an initial increase in ACE inhibition, followed by relatively stable values for the remainder of the fermentation period.

Additionally, these results suggest that ACE inhibitory activity increased from 24 h onward, coinciding with the growth of *E. faecium* ABMC-05 and the production of free amino groups during fermentation. This relationship indicates that proteolysis may have contributed to the generation of low-molecular-weight hydrolysates with ACE-inhibitory potential, although these compounds were not specifically identified by SDS-PAGE or size-exclusion chromatography. As discussed previously, bioactivity may depend more strongly on peptide sequence and amino acid composition than on the concentration of a particular protein fraction.

ACE-inhibitory peptides are generally short-chain peptides with 2–20 amino acids [87]. The presence of aromatic amino acids (phenylalanine, tryptophan, tyrosine, and proline), hydrophobic amino acids (leucine, isoleucine, and valine), and basic amino acids (arginine and lysine) at the C-terminus strongly influences ACE binding [88]. In this regard, the proteolytic system of *Enterococcus* spp., including coccolysin and glutamyl endopeptidase, preferentially hydrolyzes β-lactoglobulin. Coccolysin cleaves peptide amide bonds at the N-terminus of Leu, Phe, Tyr, and Ala, whereas glutamyl endopeptidase preferentially cleaves peptide amide bonds at the C-terminus of Glu and Asp; these amino acids are important for producing peptides with relatively high ACE inhibitory activity. Due to the negative charge of the Glu and Asp residues in the peptide sequence, they can bind to zinc ions present at the ACE active site. This interaction blocks the enzyme’s action and prevents the conversion of angiotensin I to angiotensin II [87,88].

Furthermore, several studies concur that *Enterococcus* spp. release peptides with ACE-inhibitory activity during milk and whey fermentation [68,83,89,90,91,92,93]. Specific peptide sequences (LDAQSAPLR, LKGYGGVSLPEW, and LKALPMH) from *Enterococcus* spp. Selenium compounds have been identified in whey and have been shown to inhibit ACE [89]. Interestingly, the significant inhibition observed in the whey–selenium system also correlates with selenium accumulation; in both analyses, a maximum is reached at 120 h. In this context, incorporating selenium into peptides can increase ACE inhibition. Bhuyan et al. [94] reported that replacing the L-serine residue in the Ser-Pro-Phe peptide with L-cysteine or L-selenocysteine increased ACE inhibitory activity, provided that the Phe residue at the C-terminal end of the dipeptides (Sec-Pro-Phe and Cys-Pro-Phe) was present. The authors also indicated that the presence of a free amino group in the Sec or Cys fraction can stabilize the ACE-inhibitor complex through hydrogen bonds. Although such peptides were not identified in the present study, the observed increase in ACE inhibition suggests the generation of hydrolysates with similar bioactive potential.

## 4. Conclusions

The evaluation of the growth of *Enterococcus faecium* ABMC-05 in bovine whey, with and without selenium enrichment, established that whey is a suitable substrate for the growth of this strain, that it can absorb selenium, possibly through biotransformation into organic species, and that it also produces hydrolysates with biological activity. Likewise, antihypertensive activity was more pronounced than antioxidant activity. The study represents a significant advance in the development of functional dairy products that may contain probiotics, selenium, and bioactive hydrolysates.

## Figures and Tables

**Table 1:** Selenium accumulation by *E. faecium* ABMC-05 during its growth in whey enriched with 184 mg/L of sodium selenite.

Fermentation Time (h)	Selenium Concentration in the Supernatant (mg/L)	Selenium Concentration in the Cells (mg/L)	Selenium Uptake (%)	Microbial Growth (Log CFU/mL)	Selenium Accumulation (µg Se/Log CFU)
0	31.36 ± 0.85	-	-	6.78 ± 0.03	-
24	26.05 ± 0.61	5.31 ± 0.44	16.91 ^c^	7.71 ± 0.06	0.69 ± 0.06 ^c^
48	21.97 ± 1.02	9.39 ± 0.78	29.95 ^b^	7.60 ± 0.51	1.24 ± 0.09 ^b^
72	14.93 ± 0.39	16.43 ± 1.21	52.36 ^a^	7.44 ± 0.07	2.21 ± 0.18 ^a^
96	14.58 ± 0.72	16.78 ± 1.56	53.45 ^a^	8.19 ± 0.51	2.06 ± 0.30 ^a^
120	12.87 ± 0.54	18.49 ± 1.39	58.92 ^a^	8.17 ± 0.36	2.26 ± 0.13 ^a^

All results are expressed as the mean ± standard deviation of three replicates. ^a,b,c^ Different letters denote significant differences within the same column (*p* < 0.05).

## Data Availability

The data that support the findings of this study are openly available in Zenodo at https://doi.org/10.5281/zenodo.20314231.
